# Trade and environmental pollution in Africa: accounting for consumption and territorial-based emissions

**DOI:** 10.1007/s11356-020-10328-8

**Published:** 2020-08-05

**Authors:** Samuel Adams, Eric Evans Osei Opoku

**Affiliations:** 1 Ghana Institute of Management and Public Administration, P.O. Box AH50, Achimota-Accra, Ghana; 2 University of Hamburg, Hamburg, Germany

**Keywords:** Trade, Environment, Consumption-based CO_2_ emissions, Territorial-based CO_2_ emissions, Africa, F10, F18, F64, N57, N77, Q56

## Abstract

This paper employs a recently constructed consumption-based carbon dioxide emissions data in which emissions computations are made based on fossil fuel usage domestically, in addition to emissions emanating from imports minus exports. We contrast this measure with the commonly measured territory-based carbon dioxide emissions data and examine how trade performance (split into imports, exports, and total trade) impacts these two measures of carbon dioxide. We focus on 22 sub-Saharan African countries over the period 1995–2014. Employing the system generalized method of moments, we find trade to generally have positive effect on emissions. The results are consistent across the different measures of trade and carbon dioxide emissions. The results of the paper allow us to give some policy suggestions regarding carbon dioxide emissions in sub-Saharan Africa.

## Introduction

Concerns over global warming and climate change have increased in recent times due to the negative effects on human. This has led to an extensive research agenda on the causes and impacts of environmental degradation (Khan et al. 2020). It is not surprising that Beeson (2010) describes the environment as defining public policy issue of the epoch. Many governments across the world have jumped at the policy of protecting the environment. France, for example, has made it mandatory for all listed firms to report on how their activities protect and harm the environment. The United Nations is also at the forefront of pushing the environmental protection agenda to help curtail the numerous effects of climate change especially in developing countries. Countries in the developing world are earmarked to be the hardest hit from the dangers of climate change. For Africa, in particular, the issue is more critical because though it emits the least of the greenhouse gases (GHG), it is the worst affected and the most vulnerable.

Recent climate report by the Guardian suggests that over the next eight decades, the continent will witness many dire outbreaks of heavy rainfalls (The Guardian 2019). These intense rainfalls could spark destructive floods and storms that could have enormous devastating effects on activities like farming. Moreover, these occurrences are expected to be associated with severe droughts in the course of the planting season and would affect crop production (The Guardian 2019). The report also highlighted that carbon dioxide (CO_2_) emissions were historically at their peak on earth in May 2019. The emissions levels got to 415 ppm, and scientists caution that it is probable for them to persist on this increasing trajectory for many years to come.

Increasing climate change is also anticipated to increase the possibility of violent behaviors as climate change could have political, sociological, economical, and psychological consequences (Miles-Novelo and Anderson 2019; Plante et al., 2017a, b). Temperatures all over the world will dangerously increase, and in certain parts of Africa, particularly South Africa, climate change is expected to increase violence and conflict (Chersich et al. 2019). The Foresight Africa (2019) has observed that climate change will exacerbate poverty, strain government institutions, and threaten stability in the region if nothing is done especially in the Sahel region and other unstable areas. In a special meeting at the United Nations, Inga Rhonda King, the President of United Nations Economic and Social Council, asserted that the region happens to be one of the most environmentally degraded globally. Temperature rise in the region is expected to be 1.5 times higher relative to other regions globally. West Africa, for example, is earmarked as a climate change stronghold, with the consequences expected to decrease food production and yields. This poses a serious challenge to food security. Shepard (2019) also argues that perhaps no region is suffering from climate change as the Sahel region, a region with an estimated population growth rate of 2.8% per annum and diminishing natural resources, even including water resources. For Africa, then the World Research Institute’s statement that “climate change is not a distant phenomenon-it is right here with us” is very real (Levin and Tirpak, 2018).

The African region as a whole contributed just about 2.5% to the global anthropogenic CO_2_ emissions for the period 1980–2005 (Canadell et al. 2009). Despite the fact that sub-Saharan Africa’s (SSA) emissions are least globally, they have been increasing in the last decade. Considering the consequences of rapid population increase on CO_2_ emissions and the associated adverse effects, this becomes more worrisome as SSA has one of the speediest population growth rates globally. Recent reports suggest that the region only accounts for 7.1% of the global emissions of GHGs, though it is home to 14% of global population (The Economist 2018).

In the era of globalization, trade openness is one of the key economic variables (components), aside capital flows, urbanization, and migration (O’Rouke 2002) affecting climate change. The 2017 African Trade Statistics Yearbook indicates that trade is one of the major drivers of integration and economic development in Africa (African Union 2018). The African region is poised to increase its trade volume and value as the African Continental Free Trade Area (AfCFTA) is believed to tremendously increase intra-African trade and speed the process of diversification of sources of trade (African Trade Report 2018). The main aim of the AfCFTA is to speed economic integration in the region and accelerate trade within the region. In year 2016, Africa’s intra-trade increased to 19.6% of its total trade, from 15.2% in the year 2014 to 10.3% in 2008 (World Trade Organization 2018). The AfCFTA signed in Kigali on March 2018 by 44 out of the 55 countries will fundamentally create a larger market for 55 African countries made up of about 1.2 billion people and an annual GDP of about $2.1 trillion, aggregating the present regional economic blocs into a one continental bloc (Trade and Development Report 2018). It is estimated that this move could increase intra-African trade by about 33%, employment by about 1.2%, and GDP between the range of a 1 to 3%. The successful implementation of the AfCFTA has the potential of accelerating industrialization and facilitating economic diversification and inclusion (The Foresight Africa 2019). The AfCFTA agenda is consistent with the Sustainable Development Goals, Goal 17.11, which seeks to increase developing countries’ exports and especially doubling the share of global exports of least developed countries.

The question then is how this improvement in trade is impacting on the environment? This is the issue driving this paper. The main objective of this study is to examine the effect of trade on CO_2_ emissions in SSA countries. Specifically, we find the effect of different components of trade, by examining how exports and imports of goods and services in addition to the total trade measure affect CO_2_ emissions. Previous studies have mainly focused on the total trade measure. More importantly, we find the effect of the different measures of trade on disaggregated data of CO_2_ emissions (territorial and consumption-based emissions). As the territorial-based is made up of CO_2_ emissions from domestic activities only (Boden et al. 2013; Lamb et al. 2014), the consumption-based emissions is related to the domestic use of fossil fuels in addition to the embodied emissions from imports, subtracting exports (Peters et al., 2011a, b). This is one of the first studies on SSA that examines the independent effects of total exports and total imports on the disaggregated data of CO_2_ emissions. We make a case for 22 SSA countries over the period 1995–2014. The number of countries and years for the study are influenced by data availability. Data on consumption-based CO_2_ emissions exists for 26 African countries; hence. excluding North Africa countries leaves us with 22 SSA countries.

The study continues as follows: “Related literature” presents a brief literature review on the topic. “Methodology and data” and “Results and discussion” present the methodology and the results (and discussion) of the paper respectively. “Conclusion and policy implications” concludes the paper.

## Related literature

Trade openness has been identified as one of the key ingredients of globalization besides intellectual property rights and capital flows (O’Rourke 2002), whose impact on sustainable development and particularly the environment has been contested both theoretically and empirically. Three main perspectives of trade have been identified in relation to the environment: positive impact, deteriorating (negative), and no impact. The positive impact of trade on CO_2_ emissions is associated with the fact that increased international trade leads to more production and energy consumption and consequently more pollution (scale effect) (Dean 2002). This scale effect that leads to more pollution could be augmented by the composition effect related to the changes in consumption and production patterns which ensure greater efficiency and higher output and consequently the release of more CO_2_ emissions. Put differently, the *scale effect* indicates that a greater level of economic growth (increase in per capita income) results in greater energy consumption, which leads to increases in emissions of CO_2_ (Shahbaz et al., 2019a, b). The composition effect of trade on the other hand indicates that strictness of environmental regulations would cause a move in pollution-intensive production to less developed countries (Copeland and Taylor 2003) leading to the so-called pollution havens and the development of the pollution haven hypothesis (PHH). The PHH thus emphasizes that, with an open and liberalized trade, industries that produce pollution-intensive products tend to move from advanced (rich) countries with stringent environmental standards to less developing (poor) countries with lax environmental standards, while industries producing “clean” products tend to shift towards advanced countries (Copeland and Taylor 1994). Rich countries can decrease their production of carbon-intensive products and by doing so decrease their territory-based CO_2_ emissions (Bhattacharya, Inekwe and Sadorsky 2020).

The reasoning behind the hypothesis is that environmental regulations causes cost to rise, which eventually makes exports of stringent regulations countries more expensive, compared with exports from less stringent regulations countries (Grossman and Krueger 1993; Tobey 1990). Indeed, trade liberalizations and globalization pervert the gains of countries’ policies concerning the climate change since advanced countries decrease their emissions by shifting their “dirty” industries to less developed countries (Ertugrul et al. 2016; Bilgili et al. 2016).

On the other hand, the improved growth and wealth associated with trade liberalization could trigger demand for higher quality environment and therefore more stringent enforcement of environmental policy. Additionally, an upsurge in trade flows can also make environmental quality to increase if trade speeds up better access to greener technologies in production and backs the call for environmental standards and regulations that protect the environment. Trade induces competition, and this competition can also coax countries to adopt more efficient production technologies and hence decrease carbon emissions. Thus, the technique effect associated with the transfer of knowledge and improved technological production strategies is likely to result in decrease in emissions (Shahbaz et al., 2019a, b). Runge (1993) asserts that through the utilization of cleaner technologies in production and other economic activities, trade openness is able to provide opportunities for a number of countries to attain better environmental quality by reducing CO_2_ emissions.

The effect of trade on carbon emissions therefore cannot be determined a priori*.* As noted by Grossman and Krueger (1991, 1993), the impact of trade on the environment in both developed and developing countries hinges on the sort of environmental policies they have implemented regardless of their stages of development. As Dean (2002) has noted, trade openness could have both direct and indirect impacts on emissions, and that these impacts could be either positive or negative.

Accordingly, many studies have therefore been carried out to validate the trade-carbon emissions relationship. In the recent literature, interest in disaggregated CO_2_—consumption-based and territory-based carbon emissions—is rising. In what follows, we review some of these studies. Globalization, of which trade has been the main indicator has direct consequences on the environment (Ahmed et al. 2016). Ahmed et al. (2016) find trade to affect energy consumption which in turn affects environmental degradation. Using the club convergence approach of Phillips and Sul and a total of 70 countries over the period 1990–2014, Bhattacharya et al. (2020) examines the determinants of convergence paths for both consumption-based and territorial-based carbon emission intensities. Among other results, they find that a one-unit increase in trade leads to a 1.0 (consumption-based) or 1.03 (territorial-based) increase the odds of being in the low carbon emissions intensity club. Hasanov et al. (2018) investigate the impact of trade on CO_2_ emissions for 9 oil-exporting countries. Examining the separate impacts of exports and imports and disaggregating CO_2_ emissions, consumption-based and territorial-based carbon emissions, they show that as exports and imports have statistically significant impacts of opposite signs on consumption-based CO_2_ emissions in both the long and short run, exports and imports are statistically insignificant for territorial-based CO_2_ emissions. Using a panel of 20 Asian countries over the period 1990–2013, Liddle (2018a) finds that as trade is significant for consumption-based emissions, it is not for territorial-based emissions. Specifically, he finds that exports and imports offset each other in that exports lower (negative coefficient) consumption-based emissions, whereas imports increase (positive coefficient) them. Similarly, Liddle (2018b) examines the differential impact of exports and imports on disaggregated CO_2_ emissions over the period 1990–2013 but in 102 countries. He finds results similar to Liddle (2018a); trade was significant for consumption-based emissions but not for territorial-based emissions. As exports are found to lower consumption-based emissions, imports increase them. The results are found to be consistent across various income groups. Khan et al. (2020) examine the impact of trade on disaggregated CO_2_ emissions for G7 economies and find that exports and imports are negatively and positively associated with consumption-based carbon emissions, respectively.

## Methodology and data

In this section, we describe the methodology employed in attaining the objective of the paper. Specifically, it respectively contains the following subsections; model, estimation method, and data.

### Model

In investigating the effect of trade on CO_2_ emissions, we follow empirical and theoretical literature (Hasanov et al. 2018; Liddle 2018a, b) and estimate our main empirical model as:

1 $$ {\mathrm{CO}}_{\mathrm{it}}=\sigma +{\beta}_1{\mathrm{Openness}}_{\mathrm{it}}+{\beta}_2{\mathrm{FDI}}_{\mathrm{it}}+{\beta}_3{\mathrm{GDP}}_{\mathrm{it}}+{\beta}_4{\mathrm{GDP}}_{\mathrm{it}}^2+{\beta}_5{\mathrm{Pop}}_{\mathrm{it}}+{\varepsilon}_{\mathrm{it}} $$

where *i* and *t*, respectively, denote country and time. *CO*_*it*_ is the dependent variable, and it represents a proxy for the environment and in this case represents CO_2_ emissions. *CO*_*it*_ takes any of the following variables: consumption-based CO_2_ emissions (*CO*2 _ *cons*) and territorial-based CO_2_ emissions (*CO*2 _ *terr*). *CO*2 _ *cons* is consumption-based per capita CO_2_ emissions. Consumption-based emissions are calculated based on domestic final consumption and include imports (Bhattacharya et al. 2020). Hence, they are calculated based on the domestic use of fossil fuels in addition to the embodied emissions from imports minus exports (Peters et al., 2011a, b). It is measured per year in million tons of carbon. CO2 _ terr is territorial-based emissions per capita. The territorial-based emissions consist of only emissions from domestic activities (Boden et al. 2013; Lamb et al. 2014). They are also measured per year in million tons of carbon.

*Openness*_*it*_ represents trade openness and can take any of the following: *EXP*_*it*_, *IMP*_*it*_, and *Trade*_*it*_. Where *EXP*_*it*_, *IMP*_*it*_, and *Trade*_*it*_ represent total exports (of goods and services) as a percentage of GDP, total imports (of goods and services) as a percentage of GDP, and total trade (exports plus imports) of goods and services as a percentage of GDP, respectively. *FDI*_*it*_ represents foreign direct investment (net inflows) as a percentage of GDP. *GDP*_*it*_ represents a measure of economic growth and in this case proxied by the log of GDP per capita. Talking about economic growth in relation to the environment, we cannot do away with the EKC hypothesis which asserts an inverted “U”-shaped relationship between environmental degradation (emissions) and economic growth (Grossman and Krueger 1991; Saboori et al. 2012). It implies that as the economy grows, emissions rise, but with greater growth, environmental quality sets in. To capture the curvature of the EKC, we include the squared of GDP, i.e., ${GDP}_{it}^2$ (Wang 2012). *Pop*_*it*_ represents the log of total population. *β*_1_- *β*_5_ are parameters to be estimated. *ε*_*it*_ is the error term.

### Econometric method

Considering the pollution haven hypothesis and the relationship that exists between openness and emissions, we cannot assume that openness and its component variables are strictly exogenous. As a result, we employ the system generalized method of moments (GMM) which is able to accommodate endogeneity and unobserved heterogeneity by allowing lagged internal instruments to be included in the model (see Arellano and Bond 1991; Arellano and Bover 1995; Blundell and Bond 1998; Holtz-Eakin et al. 1988). Motivation behind the use of the GMM estimator is reflected in its ability in mainly accommodating for a dearth of good external instrumental variables (Roodman 2009). We are also particularly interested in employing the GMM due to the enormous challenge of identifying, theoretically justifying, and validating external instruments (Bazzi and Clemens 2013; Durlauf et al. 2005). The GMM estimator makes use of the dynamic relationships existing in the explanatory variables. Estimations using the GMM follow two main procedures; the first procedure involves first differencing the variables which eliminates any possible bias that may result from time-invariant unobserved heterogeneity. In the second procedure, the model of interest is estimated by using the lagged values of the dependent variable(s) as instruments for the current explanatory variables. These instruments are therefore gleaned from the set of lagged dependent variable(s).

### Data

This paper covers a panel data of 22 SSA countries covering the period 1995–2014.^1^ The sampled countries and time period are limited by the availability of data for all the variables employed, particularly the CO_2_ emissions related variables.^2^ The number of countries in the sample (22) is arrived at due to the following; data on consumption-based CO_2_ emissions existed for 26 African countries, and restricting the sample to SSA, we arrived at 22 countries. With the exception of the consumption- and territorial-based CO_2_ emissions sourced/updated from Peters et al. (2011a, b) and Boden et al. (2015), all other remaining variables are obtained from the online database of the World Bank (World Development Indicators). Table 1 contains a summary of the variables and the descriptive statistics.

**Table 1:** Descriptive statistics

Variable	Description	Obs	Mean	Std. dev.	Min	Max
CO2_cons	Consumption-based CO_2_ emissions	440	21.813	60.846	0.462	360.709
CO2_terr	Territorial-based CO_2_ emissions	440	26.559	89.293	0.461	501.377
Imports	Total imports as a percentage of GDP	440	36.917	13.017	11.642	84.763
Openness	Total trade as a percentage of GDP	440	65.803	23.690	23.981	132.199
Exports	Total exports as a percentage of GDP	440	28.886	12.734	5.151	67.987
FDI	Foreign direct investment a percentage of GDP	440	2.982	3.995	−0.900	41.810
Income	Log of GDP per capita	440	6.689	0.994	4.956	9.226
IncomeSq	Log of GDP per capita squared	440	45.727	14.112	24.557	85.112
Population	Log of total population	440	16.364	1.084	13.931	18.988

## Results and discussion

This section presents and discusses the results of the paper. Before presenting the results, we test for cross-sectional dependence in the data as its existence can bias the estimates. We employ the Pesaran (2004) cross-sectional dependence test.^3^ For all the variables, the test rejected the null hypothesis of cross-sectional independence (see Table 3 in the Appendix). As cross-sectional dependence could bias the estimates, we follow Sarafidis and Robertson (2009) and perform time-specific demeaning of the data before estimation to reduce the impact of the bias. Time-demeaning the data prior to estimation successfully removes the bias from the mean group parameter (Herzer and Strulik 2017; Neal, 2015, Sarafidis and Robertson, 2009). Sarafidis and Robertson (2009) assert that one way to reduce the amount of error cross-sectional dependence in estimators (including GMM estimators) is to transform the data in terms of deviations from time averages.

Following Pesaran and Yamagata (2008), we also test for slope heterogeneity/homogeneity of the estimates of all the models we estimate. Under each dependent variable (territorial-based CO_2_ emissions and consumption-based CO_2_ emissions), we present 6 models. The results of the slope heterogeneity test (reported in Table 4 in the Appendix) show that models using territorial-based CO_2_ emissions do not suffer from slope heterogeneity bias at the 5% significance level except in the case of model 4. Nevertheless, the models presented under the consumption-based CO_2_ emissions dependent variable exhibit slope heterogeneity.^4^

In Table 2, we present results using the system GMM. Considering that the data suffer from cross-sectional dependence, we follow Herzer and Strulik (2017), Neal (2015), and Sarafidis and Robertson (2009) and demean the data prior to estimation to reduce/alleviate the effect. Before discussing the results, it is important to emphasize the validity and the consistency of the estimates which rely on the model diagnostics. The results indicate that for all the models, there is no second-order autocorrelation (see bottom of Table 2). The estimates indicate that the null hypothesis of no serial correlation between the errors cannot be rejected. This implies that the instruments emanating from the lags of the variables are valid for their current values. Also, the Sargan tests of over-identifying restrictions imply that the models are correctly specified and the instruments are valid (see the bottom of Table 2). Table 2 contains 12 models; from models 1–6, the dependent variable is territorial-based CO_2_ emissions, and from 7 to 12, the dependent variable is consumption-based CO_2_ emissions. The main independent variable is trade, and this variable is divided into three, exports, imports, and total trade of goods and services (exports plus imports of goods and services), all as a percentage of GDP. For all the estimated models (Table 2), the coefficients of the lagged dependent variables are positive and statistically significant at the 1% level. This is an indication that the dependent variables in a given year are influenced by their previous values.

**Table 2:** Effect of trade on CO2 emissions (system GMM)

	Model 1	Model 2	Model 3	Model 4	Model 5	Model 6	Model 7	Model 8	Model 9	Model 10	Model 11	Model 12
Variables	CO2_terr	CO2_terr	CO2_terr	CO2_terr	CO2_terr	CO2_terr	CO2_cons	CO2_cons	CO2_cons	CO2_cons	CO2_cons	CO2_cons
L.depvar	0.955^***^	0.954^***^	0.950^***^	0.922^***^	0.923^***^	0.916^***^	0.960^***^	0.970^***^	0.966^***^	0.946^***^	0.957^***^	0.957^***^
	(0.002)	(0.006)	(0.003)	(0.003)	(0.006)	(0.004)	(0.002)	(0.002)	(0.002)	(0.003)	(0.003)	(0.002)
Imports	0.207^***^			0.238^***^			0.121^***^			0.129^***^		
	(0.012)			(0.021)			(0.011)			(0.013)		
Exports		0.425^***^			0.431^***^			0.110^***^			0.114^***^	
		(0.018)			(0.026)			(0.017)			(0.013)	
Trade			0.219^***^			0.224^***^			0.077^***^			0.080^***^
			(0.014)			(0.012)			(0.008)			(0.007)
FDI	− 0.255^***^	− 0.213^***^	− 0.280^***^	− 0.167^***^	− 0.096^***^	− 0.178^***^	− 0.208^***^	− 0.183^***^	− 0.221^***^	− 0.165^***^	− 0.138^***^	− 0.188^***^
	(0.019)	(0.022)	(0.015)	(0.017)	(0.022)	(0.024)	(0.016)	(0.017)	(0.014)	(0.016)	(0.021)	(0.014)
Income	12.997^***^	13.448^***^	14.580^***^	− 25.830^***^	− 22.155^***^	− 20.347^***^	8.205^***^	6.557^***^	6.913^***^	− 3.410	− 6.357^***^	− 3.404^**^
	(0.330)	(0.665)	(0.499)	(2.848)	(3.197)	(5.650)	(0.453)	(0.584)	(0.543)	(2.876)	(2.059)	(1.713)
IncomeSq				3.027^***^	2.810^***^	2.762^***^				0.887^***^	0.954^***^	0.810^***^
				(0.194)	(0.217)	(0.399)				(0.150)	(0.143)	(0.100)
Population	2.575	7.639^*^	3.248	5.458	7.586	3.756	− 5.245^*^	0.150	0.187	− 6.190^*^	0.192	1.423^**^
	(3.792)	(4.269)	(3.793)	(4.824)	(5.241)	(3.788)	(2.785)	(0.318)	(0.481)	(3.298)	(0.733)	(0.555)
Constant	− 0.902	0.714	1.276	0.160	1.277	2.033	0.371	− 0.275	− 0.038	0.201	− 0.468	0.214
	(1.317)	(2.010)	(1.701)	(1.475)	(2.141)	(2.600)	(0.398)	(0.400)	(0.464)	(0.439)	(0.536)	(0.570)
Observations	418	418	418	418	418	418	418	418	418	418	418	418
No. Countries	22	22	22	22	22	22	22	22	22	22	22	22
AR2	− 1.103	− 1.104	− 1.112	− 1.119	− 1.119	− 1.130	1.181	1.255	1.221	1.191	1.269	1.245
AR2(P)	0.270	0.270	0.266	0.263	0.263	0.258	0.238	0.210	0.222	0.234	0.205	0.213
Sargan test	19.925	19.064	20.862	20.504	20.535	19.517	15.995	20.900	19.726	14.961	19.961	19.184
Sargan(P)	0.981	0.987	0.972	0.976	0.975	0.984	0.998	0.972	0.982	0.999	0.981	0.986
Instrs.	41	41	41	42	42	42	41	41	41	42	42	42

Standard errors in parentheses. ^*^p < 0.10, ^**^p < 0.05,^***^ *p* < 0.01. Dependent variables: CO2-terr and CO2-cons are territorial and consumption-based CO2 emissions, respectively. L.depvar is the lag of the dependent variable

The results indicate that openness as measured by total trade (as a percentage of GDP) has a statistically positive coefficient (1% level) for both the consumption-based and territorial-based CO_2_ emissions estimations (see models 3 and 9 of Table 2). Similarly, in models 6 and 12 when the models were augmented by GDP squared, trade is still positive and statistically significant.

Using exports and (as a percentage of GDP) to proxy for openness, the results still show positive and statistically significant coefficients (models 2 and 5 of Tables 2) irrespective of the dependent variable. Exports still exhibit statistically positive coefficients when the models are augmented with GDP squared (models 8 and 11 of Table 2). Imports (as a percentage of GDP) also show positive and statistically significant coefficients (models 1 and 7 of Table 2) even when the models are augmented with GDP squared. The results generally indicate that trade (irrespective of the measure) leads to increase in CO_2_ emissions (regardless of the measure).

To further investigate the impact of trade on the environment, we split total trade to its components: exports and imports. The results of both exports and imports are similar to that of total trade irrespective of the dependent variable (models 2 and 7 of Table 2). Since trade is divided into exports and imports, the expectation is that exports will reduce and imports will increase consumption-based emissions (Hasanov et al. 2018). The results show the coefficients of exports in the consumption-based emissions to be positive and statistically significant (see models 8 and 11 of Table 2), implying that increase in exports increases consumption-based emissions. The results of exports are contrary to the expectation (Hasanov et al. 2018). The consumption-based emissions are calculated based on domestic final consumption and includes imports but excludes exports (Bhattacharya et al. 2020).

However, the positive impact of exports on consumption-based emissions may be explained by the fact that products that are exported require the use of machinery and other products that are imported to especially facilitate processing or production of the goods to be imported. To our expectation, the results indicate that imports have positive and statistically significant coefficients (see 7 and 10 of Table 2), implying that increase in imports increases consumption-based CO_2_ emissions. Consumption-based emissions include embodied emissions from imports, as a result increase in imports will increase their emissions (Peters et al., 2011a, b). Imported goods and services form a great chunk of the of the total consumption of developing countries; they import a substantial amount of intermediate and final goods to consume domestically, and as a result, consumption-based CO2 emissions increase (Hasanov et al. 2018). The results are consistent with Hasanov et al. (2018) and Liddle (2018a, b).

Regarding the estimations using the territorial-based emissions as the dependent variables, we find the results to be consistent with those using consumption-based emissions. Both exports and imports have positive and statistically significant coefficients. Since the territorial-based emissions are made up of CO_2_ emissions from domestic activities including production for exports (Boden et al. 2013; Lamb et al. 2014), the results of the exports variable meet our expectation as increase in exports increases territorial-based emissions. The results of imports defy our expectation. Nevertheless, in cases where imported products have to be reprocessed or reproduced in the domestic economy importing them, increase in such imports will add up to the territorial-based emissions in that economy. Our results are largely contrary to some studies that have found trade not to matter for territorial-based emissions (Liddle 2018a, b; Hasanov et al. 2018; Khan et al. 2020).

The results of the study indicate that regardless of the measure of trade or emissions, increase trade is associated with increased emissions. This implies that trade is harmful to environmental quality (as they lead to increase in both territorial- and consumption-based CO_2_ emissions). Generally, the results buttress the argument of the pollution haven hypothesis. The pollution haven hypothesis suggests that with globalization and the opening up of countries for trade, multinational firms in more developed countries are bound to move their “dirty” production to developing or poor countries. This is the case as developing countries have lax environmental regulations and are in dire need of trade, considering the many benefits that come with it. In SSA, the structural and economic recovery programs of the 1980s saw the opening up of more countries for trade. The results largely tell that total trade has not contributed in improving environmental quality. This outcome is generally in consonance with a number of studies (see for example, Bento and Moutinho 2016; Jebli et al. 2019; Zeng et al. 2019; Opoku and Boachie, 2020).

In relation to the other control variables, the results indicate that irrespective of the dependent variable, the FDI variable is found to be consistently negative and statistically significant. This implies that increase in FDI is likely to cause environmental quality to improve. This leans support to the pollution halo hypothesis (Kahia et al. 2019; Huang et al. 2019; Jebli et al. 2019), which argues that multinational firms possess superior technologies and as a result are capable of engaging in green investments and activities that do not hurt the environment (Doytch and Uctum 2016; Wang 2017). Regardless of the dependent variable, the results generally show population to have positive coefficients howbeit statistically insignificant.

For economic growth (GDP), the results indicate positive and statistically significant coefficients regardless of the dependent variable, indicating that rising economic growth can hurt the environment. A rise GDP implies a rise in the income level of the countries in the sample. A rise in income will increase economic activity. Individuals, firms, and governments in these countries can demand more goods whose production and consumption result in increase in CO_2_ emissions (Hasanov et al. 2018, Liddle, 2018a). Khan et al. (2020) assert that increase in economic activities as a result of increase in GDP increases energy consumption hence causing CO_2_ emissions to rise. Nevertheless, in accounting for the EKC by including the square of GDP, we find contrary results. We find GDP having negative coefficients with the squared GDP having positive coefficients. In contrast to the underpinnings of the EKC hypothesis, the results indicate that at the initial levels of growth, growth is not harmful to the environment; however, it becomes harmful at higher stages of the growth expedition. Following Hasanov et al. (2018), we argue that this outcome may be as a result of the countries in our sample. The countries in the sample are developing countries, and they will continue to grow in the long run especially in industrial development which has not fully taken place in these African countries. With this, higher CO_2_ emission is expected with greater increase in GDP. The results of the study affirm the findings in many empirical studies that the EKC hypothesis usually does not hold for developing countries (Hasanov et al. 2018; Hasanov et al. 2019). The countries in our sample are developing countries and have long way to go to have the economic, institutional, and environmental systems in which rise in income will result in reduction in CO_2_ emissions (Hasanov et al. 2019). Harbaugh et al. (2002) assert that the evidence for EKC is less robust than previously claimed.

## Conclusion and policy implications

The results of the paper allow us to give some policy implications regarding CO_2_ emissions in SSA. The observation is that trade and its components generate positive impact on both territorial and consumption-based CO_2_ emissions. The results, hence, imply that trade, regardless of how it is measured, leads to environmental degradation in the form of increased CO_2_ emissions. The results are in line with the hypothesis that openness could pollute developing countries. Trade is very important to countries in Africa. Despite the fact that almost all economies in Africa rely mainly on exports, these economies are also very import-dependent. The region is a net-importer of consumables. As the region’s domestic activities of trade (export production) could increase the emissions of territorial based CO_2_, its imports increase consumption-based CO_2_ emissions. As both exports and imports are bound to happen in all African countries, CO_2_ emissions are also bound to happen. Governments and policymakers must therefore be conscious of the emissions capabilities of trade. This is important considering the effect of CO_2_ emissions on climate change. Climate change is one of the greatest challenges societies all over the world (especially African countries) are contending with, and there is mounting interest in reducing CO_2_ emissions around the globe.

Deliberate attempts by governments and policymakers in Africa are presently pressing considering the initiation of the African Continental Free Trade Area (AfCFTA). The AfCFTA, which came into force in 2019, requires members to remove tariffs from 90% of goods, allowing free access to commodities, goods, and services across the African region. It is aimed at expanding intra-African trade across the region. With the AfCFTA, trade (both exports and imports) is bound to increase within the region, and hence if stringent environmental measures are not put in place, environmental degradation in the form of carbon emissions is bound to rise. As a result, environmental policymakers have to execute and monitor strict environmental regulatory framework to effectually counter the deteriorating impact that this trade openness may come with. The AfCFTA secretariat can also come up with environmental regulations for its members to abide with. The negative effect of trade should not lead to less trade to reduce pollution, but rather the countries should embark on environmental assessment mechanisms which can in the long run make the positive impact of trade on environmental quality stronger. Improving the quality of the trade basket can be more productive than an increase in the volume of trade.

The results indicate that trade policy in the SSA region should be directed at attracting FDI in high-tech industries in the long run and also those that could help in production of renewable energy options. A major option for SSA countries is to motivate a greater set of both domestic and foreign investment so as to produce a higher scale of output and at the same time to achieve sustainable environmental quality. The way forward is for SSA countries to implement stringent regulatory frameworks that balance investment policies and environmental standards such that environmental friendly FDI will be attracted.

Realistically, every research has some limitations. Obviously, not all variables can be studied at the same time, which suggests the possibility of omitted variables. Some of the models estimated here failed to pass the slope homogeneity test, drawing a little caution to the interpretation of the results. Finally, this is a regional study of 22 SSA countries that assumes similar economic, political, and sociocultural characteristics. Future research should focus on more country-specific studies to provide more information on the trade/globalization-CO_2_ emissions relationship.

## Appendix

**Table 3:** Cross-sectional dependence tests

Variable	CD-test	*p* value	Abs (corr)
CO2_cons	45.590***	0.000	0.751
CO2_terr	42.630***	0.000	0.720
Imports	13.440***	0.000	0.323
Openness	9.940***	0.000	0.337
Exports	3.960***	0.000	0.341
FDI	12.790***	0.000	0.296
Income	59.520***	0.000	0.876
IncomeSq	59.960***	0.000	0.882
Population	67.250***	0.000	0.989

NB: ****p* < 0.01

**Table 4:** Slope heterogeneity test (Pesaran and Yamagata (2008))

Models	CO2_terr		CO2_cons	
	Test	*p* value	Test	*p* value
Model 1	−1.723	0.085	4.107***	0.000
Model 2	−1.602	0.109	3.809***	0.000
Model 3	−1.818	0.069	3.702***	0.000
Model 4	2.204**	0.028	6.175***	0.000
Model 5	−0.01	0.989	3.604***	0.000
Model 6	−0.375	0.708	4.035***	0.000

NB: ***p* < 0.05, ****p* < 0.01

## Footnotes

1 The list of countries is as follows: Benin, Botswana, Burkina Faso, Cameroon, Cote d’Ivoire, Ghana, Guinea, Kenya, Madagascar, Malawi, Mauritius, Mozambique, Namibia, Nigeria, Rwanda, Senegal, South Africa, Tanzania, Togo, Uganda, Zambia, and Zimbabwe.

2 In order to attain balanced panel for all the variables employed and to include as many countries as possible, we restrict end of the sample to 2014.

3 This test is implemented in Stata using the command xtcd.

4 The results hold for the main data and the time-specific demeaned data. Considering the existence of slope heterogeneity for some of the models, estimators robust (such as CS-ARDL, AMG or CCEMG) to this bias are prudent to be used for these models. However, none of these estimators worked for our data. Besides, these estimators are applied to static panel models with strictly exogenous regressors. As a result, we proceed to estimate our main results with the GMM on time-specific demean data.
