# Correction to: Trade and environmental pollution in Africa: accounting for consumption and territorial-based emissions

**DOI:** 10.1007/s11356-021-15429-6

**Published:** 2021-07-24

**Authors:** Samuel Adams, Eric Evans Osei Opoku

**Affiliations:** 1 Ghana Institute of Management and Public Administration, P.O. Box AH50, Achimota Accra, Ghana; 2 University of Hamburg, Hamburg, Germany

**Correction to: Environ Sci Pollut Res (2020) 27:44230–44239**

10.1007/s11356-020-10328-8

The article Trade and environmental pollution in Africa: accounting for consumption and territorial-based emissions written by Samuel Adams and Eric Evans Osei Opoku, was originally published electronically on the publisher’s internet portal on 5 August 2020 without open access. With the author(s)’ decision to opt for Open Choice the copyright of the article changed on 2 July 2021 to © The Author(s) 2021 and the article is forthwith distributed under a Creative Commons Attribution 4.0 International License, which permits use, sharing, adaptation, distribution and reproduction in any medium or format, as long as you give appropriate credit to the original author(s) and the source, provide a link to the Creative Commons license, and indicate if changes were made. The images or other third party material in this article are included in the article’s Creative Commons license, unless indicated otherwise in a credit line to the material. If material is not included in the article’s Creative Commons license and your intended use is not permitted by statutory regulation or exceeds the permitted use, you will need to obtain permission directly from the copyright holder. To view a copy of this license, visit http://creativecommons.org/licenses/by/4.0.

The Original article has been corrected.

